# Genomic evidence of demographic fluctuations and lack of genetic structure across flyways in a long distance migrant, the European turtle dove

**DOI:** 10.1186/s12862-016-0817-7

**Published:** 2016-11-07

**Authors:** Luciano Calderón, Leonardo Campagna, Thomas Wilke, Hervé Lormee, Cyril Eraud, Jenny C. Dunn, Gregorio Rocha, Pavel Zehtindjiev, Dimitrios E. Bakaloudis, Benjamin Metzger, Jacopo G. Cecere, Melanie Marx, Petra Quillfeldt

**Affiliations:** 1 Department of Animal Ecology & Systematics, Justus Liebig University Giessen, Heinrich-Buff-Ring 82, Giessen, 35392 Germany; 2 Fuller Evolutionary Biology Program, Cornell Lab of Ornithology, 159 Sapsucker Woods Road, Ithaca, NY 14850 USA; 3 Department of Ecology and Evolutionary Biology, Cornell University, 215 Tower Road, Ithaca, NY 14853 USA; 4 Unité Avifaune Migratrice, Office National de la Chasse et de la Faune Sauvage, Villiers en Bois, Chizé 79360 France; 5 School of Life Sciences, University of Lincoln, Joseph Banks Laboratories, Lincoln, LN6 7TS UK; 6 Department of Agro-forestry Engineering, University of Extremadura, Avda. Virgen del Puerto 2, Plasencia, Cáceres 10600 Spain; 7 Bulgarian Academy of Sciences, Institute of Biodiversity and Ecosystem Research, 2 Gagarin Street, Sofia, 1113 Bulgaria; 8 Aristotle University of Thessaloniki, School of Forestry and Natural Environment, PO Box 241, Thessaloniki, 541 24 Greece; 9 Birdlife Malta, Triq Abate Rigord, Ta’ Xbiex, XBX 1120 Malta; 10 Instituto Superiore per la Protezione e la Ricerca Ambientale, Via Ca’ Fornacetta 9, Ozzano Dell’Emilia, (BO) 40064 Italy

**Keywords:** Population genetic structure, Demography, Migratory birds, Genomics, Conservation, Paleoclimatic niche modelling, Climate change

## Abstract

**Background:**

Understanding how past climatic oscillations have affected organismic evolution will help predict the impact that current climate change has on living organisms. The European turtle dove, *Streptopelia turtur*, is a warm-temperature adapted species and a long distance migrant that uses multiple flyways to move between Europe and Africa. Despite being abundant, it is categorized as vulnerable because of a long-term demographic decline. We studied the demographic history and population genetic structure of the European turtle dove using genomic data and mitochondrial DNA sequences from individuals sampled across Europe, and performing paleoclimatic niche modelling simulations.

**Results:**

Overall our data suggest that this species is panmictic across Europe, and is not genetically structured across flyways. We found the genetic signatures of demographic fluctuations, inferring an effective population size (*Ne*) expansion that occurred between the late Pleistocene and early Holocene, followed by a decrease in the *Ne* that started between the mid Holocene and the present. Our niche modelling analyses suggest that the variations in the *Ne* are coincident with recent changes in the availability of suitable habitat.

**Conclusions:**

We argue that the European turtle dove is prone to undergo demographic fluctuations, a trait that makes it sensitive to anthropogenic impacts, especially when its numbers are decreasing. Also, considering the lack of genetic structure, we suggest all populations across Europe are equally relevant for conservation.

**Electronic supplementary material:**

The online version of this article (doi:10.1186/s12862-016-0817-7) contains supplementary material, which is available to authorized users.

## Background

Climate oscillations during the Quaternary strongly affected organismic evolution worldwide (e.g. [1–3]). The responses of different species to these oscillations have been diverse, and dependent on their environmental requirements and adaptations [4, 5]. In Europe, for example, most species that are adapted to warm-temperate climates underwent southward range shifts and reductions in their abundance during glacial periods, recovering their northern distributions and numbers during interglacial periods [6–8]. Similarly, current global warming and human activities are also affecting the evolution and distribution of living organisms worldwide (e.g. [9, 10]). Long distance migratory bird species are particularly sensitive to global climate change; their migration routes and timing have shifted drastically during the past decades [11, 12], mainly as a consequence of phenological responses to the rising temperatures [13]. Moreover, long distance migrants have suffered stronger population declines than other bird species, because their survival depends on the quality of habitats that are often geographically distant (i.e. breeding, stopover and wintering territories) [11, 14, 15].

Around 2 billion birds migrate between Europe and Africa each year [16], crossing the Mediterranean Sea and the Sahara Desert. Even though migration of some species occurs broadly throughout the Mediterranean Sea, higher numbers of birds migrate over its eastern and western edges [17], thus reducing over-sea flying distances. As a consequence, alternative flyways have arisen in some species (e.g., White storks, *Ciconia ciconia*, [18]; Montagu’s harrier, *Circus pygargus*, [19]. Similar strategies have been observed in other long distance migratory birds across the world (e.g. North American’s Swainson’s thrush, *Catharus ustulatus*, [20]; the Asian greenish warbler, *Phylloscopus trochiloides*, [21]). In some cases, such alternative flyways might be mirrored in the breeding grounds in the form of migratory divides, defined as narrow regions of contact between populations with different migratory pathways [22]. The presence of alternative flyways and migratory divides has been postulated as a mechanism promoting intraspecific genetic diversification [23].

**Fig. 1:**
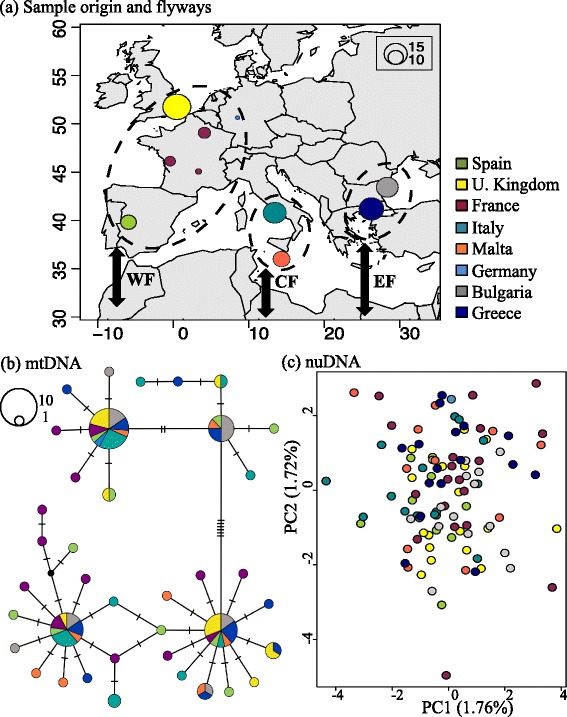
Sampling localities and population genetic structure from mitochondrial and nuclear data. Legend: **a** Coloured circles indicate sample origin; circle sizes are scaled to sample size. Dashed ovals group the sampling localities according to the three main flyways (represented with *arrows*): western, central and eastern (WF, CF and EF, respectively). **b** Median joining network based on cytochrome *b* haplotypes (mtDNA), hatch marks on the lines connecting circles indicate mutational steps. **c** Principal component analysis based on SNP data (nuDNA)

The European turtle dove, *Streptopelia turtur* (hereafter turtle dove), is the only columbid that takes part in the African-European migration system [16]. The species is warm-climate adapted and requires patchy areas composed by shrubby woodlands and open grasslands to breed [24]. The turtle dove breeds across the western Palearctic and spends winters in the sub-Saharan African region, in latitudes between 10°N and 20°N [25]. Mark-recovery data suggests that turtle doves use three main flyways during their spring and autumn journeys [26]. Birds that breed in western Europe fly over the Iberian Peninsula, those breeding in central Europe fly through Italy and Malta, and eastern European breeders fly over Greece (Fig. 1a). Despite being an abundant species, the turtle dove has been undergoing a long-term demographic decline, and as a result is considered a vulnerable species throughout its European distribution [27]. Both the role of the alternative migratory flyways on genetically structuring turtle dove populations, and how the long-term demographic decline has impacted the species’ genetic diversity remain unknown.

Here we studied the demographic history and population genetic structure of the turtle dove across Europe. We performed a genome wide scan using double digest restriction-site associated DNA sequencing (ddRADseq) and obtained mitochondrial DNA sequences (cytochrome b). Demographic history analyses were complemented with niche modelling analysis to test if variations in the effective population size are correlated with past changes in the availability of suitable habitat. Based on the ecological requirements of our focal species, we expected to find evidence of large effective population sizes during warm interglacial periods, followed by small effective sizes during cold glacial periods. We would expect to observe a similar trend in the availability of suitable habitat. We also hypothesized that the current demographic decline in the turtle dove would be strong enough to show the genetic signature of a decrease in its effective population size. Finally, we tested whether the alternative flyways described for the turtle dove actually correspond to migratory divides promoting intraspecific genetic diversification. In that case we expect to find three genetically differentiated groups associated to each flyway (i.e. East, Central and West).

## Methods

### Sampling scheme

We analysed samples from 104 adult individuals, representing the three main migratory flyways of the turtle dove (Fig. 1a). The western flyway (WF) was represented with samples from the United Kingdom (*n* = 18, breeding season), Spain (*n* = 10, breeding/migrating), Germany (*n* = 1, breeding season) and France (*n* = 19, breeding); the Central flyway (CF) with samples from Italy (*n* = 15, migrating) and Malta (*n* = 11, migrating); and the Eastern flyway (EF) with samples from Bulgaria (*n* = 14, breeding/migrating) and Greece (*n* = 16, breeding/migrating). Sampling took place between 2011 and 2014. Tissue samples were taken from wings of hunted individuals, and blood samples were taken from the brachial vein of individuals either caught at their breeding sites or during migration while on a flyway. Tissue samples were preserved in ethanol, and blood samples were preserved either in ethanol or on FTA cards (Whatman International Ltd., Maidstone, UK). A table showing more details of the analysed samples can be found in the additional files section (Additional file 1).

### Laboratory methods

Genomic DNA was isolated using the DNeasy blood and tissue kit (Qiagen, CA) and quantified with a Qubit fluorometer (Invitrogen, CA). Double-digest restriction-site associated DNA markers (ddRADtags) were generated following Peterson et al., [28]. We also obtained 95 sequences of the cytochrome *b* mitochondrial gene, 892 base pairs long (GenBank accession numbers: KU588290-KU588384). Primers used were specifically designed for this study: F_cytb_St: 5′-TGATAACTCAAATCCTAACTGGTC-3′ and R_cytb_St: 5′-TTGTTTTCTAGGGCTCCGAT-3′. Further details on the ddRADtags and cytochrome *b* amplification laboratory methods are provided in the additional files section (Additional file 2).

### RAD-sequence pre-processing and single nucleotide polymorphism (SNP) calling

#### Filtering and quality control

The samples were sequenced on two Illumina HiSeq 2500 lanes that produced a total of ~304 million, 151 bp single-end reads. After assessing quality using FASTQC (http://www.bioinformatics.babraham.ac.uk/projects/fastqc/), we trimmed the final 29 bp to discard lower-quality base calls using FASTX-Toolkit (http://hannonlab.cshl.edu/fastx_toolkit/). We filtered reads with at least a single base call with a Phred quality score below 10 (90 % call accuracy), and/or more than 5 % below a Phred score of 20 (99 % call accuracy). We demultiplexed reads with the process_radtags program from the STACKS v1.20 [29] bioinformatics pipeline. We discarded reads that did not contain an SbfI cut site or a unique P1 barcode in the 5′ end, as well as those with adapter contamination in the 3′ end. Finally, because the barcodes used for demultiplexing ranged between 5 and 7 bp, we trimmed all reads to 115 bp, the length of the shortest sequences once barcodes were removed.

#### Reference-based RAD loci assembly and variant calling

We obtained 1.03 ± 0.46 million, 115 bp quality-filtered sequences per individual, which were aligned to the *Columba livia* genome (assembly 1.0) with BOWTIE2 v2.2.2 [30]. The average alignment rate was 85.7 %. We conducted a referenced-based assembly using the pstacks/cstacks/sstacks pipeline controlled by the ref_map script in STACKS, followed by the rxstacks error correction module and a final iteration of cstacks/sstacks. This pipeline will identify a locus when a minimum coverage is reached: set to 20 with the m parameter. A catalogue of all loci is generated, and we allowed up to five differences among aligned loci of different individuals (parameter n). The error correction module examines SNP and haplotype calls across all individuals and removes loci that match more than one locus in the catalogue (presumably from repetitive areas of the genome), eliminates excess haplotypes that are uncommon and thus probably erroneous, and eliminates loci below a user established likelihood threshold (set to −200 based on the distribution across all loci). Once errors are corrected a new catalogue is generated and SNPs are identified. The pipeline produced a catalogue consisting of 18,026 loci. We exported SNP and sequence data for downstream analysis using the program *populations*, retaining loci that were present in every sampling locality, had less than 20 % missing data and a coverage of at least 20x. These settings produced 3,012 RAD loci, 2,934 of which had at least one SNP. When a RAD locus had more than one SNP, we selected the first one for downstream analyses to avoid using tightly linked SNPs.

### Analysis of population genetic structure

#### mtDNA analyses

Summary statistics of levels of polymorphism (i.e. haplotype and nucleotide diversity) were obtained with DNASP v5.10.1 [31]. We obtained a median joining haplotype network, using the software POPART [32]. Hierarchical analyses of molecular variance (AMOVAs) were performed in ARLEQUIN v3.5.2.2 [33], with significance values obtained after 10,000 permutations. We tested if grouping individuals by migration flyway explained significant levels of molecular variance, a signal of genetic differentiation. We tested for the possibility of three genetically differentiated groups (i.e. flyways), a Western group including samples from United Kingdom, France, Germany and Spain; a Central group containing the samples from Italy and Malta, and an Eastern group with the samples from Bulgaria and Greece.

#### SNP analyses

Genetic structure was investigated using two kinds of approaches; model and non-model based methods. For a model-based approach we employed the variational Bayesian inference method implemented in fastSTRUCTURE [34], to investigate the most likely number of genetic clusters (*K*) that better explained our data. Variational Bayesian inference aims to solve the problem of inference as an optimization rather than a sampling problem, a key advantage of this method is that it runs faster while achieving similar levels of accuracy than other available algorithms [34]. We performed a first exploratory run using the *simple prior* testing from *K* = 1 to *K* = 10. Afterwards, three independent runs with different starting random seeds were performed with the *logistic prior,* testing from *K* = 1 to *K* = 3; the usage of the latter prior is advised when genetic structure is difficult to determine or weak [34]. During both runs all other settings were left as default. Mean admixture proportions (*Q*-values) for the value of *K* that better explained our data were graphed with DISTRUCT v1.1 [35]. For non-model based methods we performed a Principal Component Analysis using the function *dudi.pca*, from the ADE4 R package [36]. An AMOVA was conducted with ARLEQUIN v3.5.2.2 [33] following the grouping scheme described above (see mtDNA analyses). We also searched for loci under selection among the groups described above using BAYESCANR [37] included in the R package PopGenome [38].

### Demographic history

We searched for signatures of past population expansion or contraction in the turtle dove effective population size (*Ne*). The *Ne* is defined as the population size that is evolutionary relevant (i.e., the number of breeding individuals) [39]. We used DNASP v5 [31] to investigate our mtDNA data set, calculating Fu’s *F*_*s*_ and Tajima’s *D*; the significance for each test was estimated after 1,000 permutations.

We also explored the demographic history of the turtle dove by performing an Extended Bayesian Skyline plot (EBSP), as implemented in BEAST version 1.8.2 [40]. We did not find evidence of population genetic structure (Fig. 1b and c), and thus were able to pool all our samples into one population. Because of the computationally intensive nature of this analysis, we conducted runs including only 30 loci per individual. We used our cytochrome *b* data and sequences from 29 randomly selected nuclear loci. All partitions were unlinked and we implemented a strict clock model, with a substitutions rate of 2.1 % per million years as estimated by Weir and Schluter [41] for the cytochrome *b* gene in birds. We ran the analysis using the “Coalescent: Extended Bayesian Skyline” tree prior for 100 million generations, sampling every 4,000. We inspected the results in Tracer version 1.6 [42], checking for convergence, sufficiently large effective sample sizes for parameter estimates and discarding the initial 10 % of samples as burn-in. This analysis was run three times using independent sets of 29 randomly selected nuclear loci in combination with the cytochrome *b* sequences; all three runs produced similar results.

**Fig. 2:**
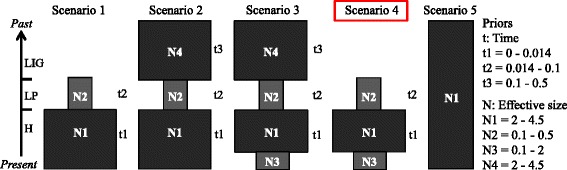
Demographic scenarios tested with DIYABC. Legend: Schematic representation of the five demographic scenarios tested and values for the priors used in DIYABC simulations. *N* represents effective population sizes (*Ne*) in millions of individuals and *t* indicates time in million years before present. Timeline from present to past indicates Holocene (H), late Pleistocene (LP) and last interglacial (LIG)

An Approximate Bayesian Computation (ABC) analysis was performed using DIYABC v2 [43]. ABC methods bypass exact likelihood calculation by using summary statistics and massive simulations, allowing them to handle large data sets [44]. Thus, we included the complete SNP dataset (2954 loci) for all genotyped individuals (104) and pooled our samples into a single population (as previously explained for the EBSP). We compared five alternative demographic scenarios, with different combinations of *Ne* variation in time (*t*), see Fig. 2 for a schematic representation of the scenarios. All priors were set as normally distributed, time (t) expressed in million years before present and effective population size (N) in million individuals (Fig. 2). Priors N1, N2, t1 and t2 were informed by the EBSP (see results). Prior N4 at t3 was set equal to N1 based on a similar availability of suitable habitat estimated for the last interglacial (LIG) and the present (see results). Prior N3 was based on the demographic decline reported for the turtle dove [27]. Simulated scenarios were generated using all the available summary statistics in DIYABC for a single population analysis based on SNPs: proportion of monomorphic loci, mean gene diversity across polymorphic loci, variance of gene diversity across polymorphic loci and mean gene diversity across all loci. Runs consisted of 5 ×10^6^ simulations (1 million simulations per tested scenario). The PCA function embedded in DIYABC was used to test the goodness-of-fit of the observed data against that generated by the simulated scenarios. The relative posterior probability was then estimated for each scenario with the *direct* and *logistic regression* approaches, on 0.1 and 1 % respectively, of the simulations closest to the observed data set. Finally, we estimated the posterior distribution of parameters for the most likely scenario; we assumed a generation time of one year for the turtle dove [45] to scale the estimated parameters.

### Niche modelling

The realized niche of the present distribution of the turtle dove was modelled and extrapolated using MaxEnt 3.3.3 k (maximum entropy; [46]). We combined data obtained from GBIF (Global Biodiversity Information Facility, http://www.gbif.org/), with the Bioclim data sets (http://worldclim.org/) for the present, the Mid-Holocene (MH, 6000 ybp), the Last Glacial Maximum (LGM, 22,000 ybp), and the last interglacial (LIG; ~120,000–140,000 ybp). MaxEnt t is a program for modelling ecological niches from presence-only species records (further details in Additional file 2).

## Results

### Summary statistics and population genetic structure

Cytochrome *b* sequences were highly polymorphic, with 73 segregating sites and 40 haplotypes found among 95 turtle dove individuals (haplotype diversity (*Hd*): 0.92; nucleotide diversity (*π*): 0.0075). Our median joining network suggested two main haplogroups, differentiated by six mutational steps (Fig. 1b). However, the observed haplogroups did not cluster samples by migration flyway or their geographic origin. Moreover, haplotypes from individuals sampled in the same locality appeared scattered across the network (Fig. 1b). Similarly, the AMOVA based on mtDNA sequences did not show evidence that migration flyways explain population genetic structure in the turtle doves. The highest percentage of the variance was explained by the components “among individuals, within population” and “within individuals” (Additional file 3).

SNP-based analyses with fastSTRUCTURE -using the *simple prior-* suggested that the number of components used to explain the genetic structure in our data (*K* ^*^_Øc_) and the model complexity that maximizes the marginal likelihood (*K* ^*^_ε_) were both 1. When the *logistic prior* was used, *K* ^*^_Øc_ was still 1, however *K* ^*^_ε_ = 3. Even though *K* = 3 was suggested with the latter prior, the greatest proportion of the ancestry of all individuals was explained by only one cluster (mean *Q* = 0.999) (shown in Additional file 4). Hence, *K* = 1 seems to be the most likely partition to explain genetic structure in our data (Additional file 4). The AMOVA result based on SNPs resembled the results obtained for the mtDNA sequences, with the “among groups” component explaining the lowest percentage of the variance when testing for the differences among flyways (Additional file 3). The PCA based on SNP data did not show any clustering of individuals (Fig. 1c). Finally, we did not find loci under selection in BayescanR (*p* > 0.05).

### Demographic history and niche modelling

**Fig. 3:**
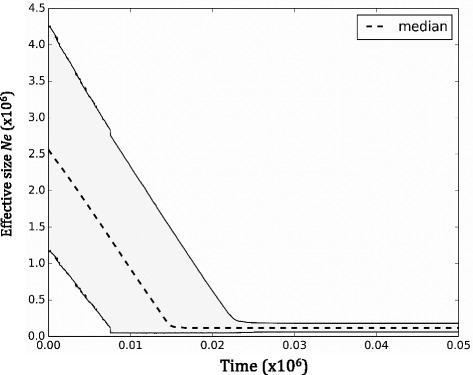
Extended Bayesian Skyline ploy analysis. Legend: The dashed line indicates the median effective population size and the grey shaded area the 95 % HPD interval

The mtDNA dataset showed signatures of a recent demographic expansion in the turtle dove population: *F*_*s*_ = −20.95 (*p* = 0.001) and *Tajima’s D* = −1.70 (*p* = 0.02). The EBSP analysis that combined mtDNA sequences with 29 randomly selected nuDNA loci also supported a population expansion. Based on our assumption of molecular clock calibration for cytochrome *b* (2.1 per million years), this expansion started ~14,000 years before present (Fig. 3).

Our DIYABC analysis supported a compatible, yet more complex demographic scenario than that suggested by the EBSP. From the five simulated scenarios, scenario four (Fig. 2) showed the best goodness-of-fit to the observed data (Additional file 5) and had the highest posterior probability, 0.99 (CI 95 % = 0.97–1) when using the *direct estimation* method and 1 (CI 95 %–1–1) with the *logistic regression* method (Additional file 6). Regardless of the method used, the confidence intervals estimated from the competing scenarios did not overlap. Scenario four assumes that the turtle dove population suffered two variations in its effective population size. The *Ne* during the mid-late Pleistocene (~78,300 ybp) was estimated to be ~84,400 individuals. Following a demographic expansion during the early-mid Holocene (~7,600 ybp), the population reached an estimated *Ne* of ~3,160,000 doves. Finally, we found evidence of a recent contraction that started between the mid-Holocene and the present, resulting in a current *Ne* estimate of ~172,000 individuals (see Table 1 for more details on the estimated parameters). It is possible that the recent contraction in the *Ne* was not detected by the EBSP analysis because of lack of statistical power in the smaller dataset (30 loci). None of our analyses recovered evidence of a large *Ne* during the last interglacial (~120–140,000 ybp).

**Table 1:** Demographic parameters inferred with DIYABC

Parameters	Mean	Quantiles (0.025–0.975)
Late Pleistocene (t2)	78,300	37,100–99,300
Mid Holocene (t1)	7,600	3,600–9,920
Late Pleistocene *Ne* (N2)	84,400	53,800–122,000
Mid Holocene *Ne* (N1)	3,160,000	2,040,000–4,430,000
Present *Ne* (N3)	172,000	107,000–432,000

Caption: Mean estimates and quantiles of the posterior distribution for the demographic parameters inferred for scenario four using DIYABC

**Fig. 4:**
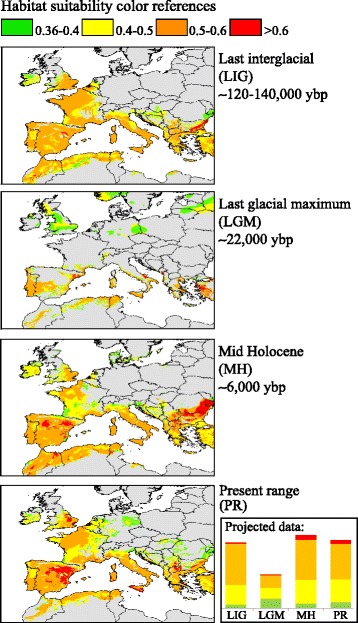
MaxEnt niche modelling analysis. Legend: Niche modelling based on the present occurrence data for the turtle dove and extrapolations into past climate models, using the global climate model MIROC-ESM. Higher values (and warmer colours) denote more suitable areas. A graph comparing the total area of different suitability classes (projected data) during different times is given in the bottom right corner

MaxEnt models achieved a good fit to the data of the present distribution (AUC value of 0.71 ± 0.01 SD). Bio1 (Annual mean temperature) was identified as the most important parameter (Percent contribution, PC: 47.9), and also achieved the highest importance in the permutation test (Permutation importance, PI: 45.5). Other key parameters were Bio18 (Precipitation of Warmest Quarter, PC: 24.2, PI: 22.3) and Bio12 (Annual Precipitation, PC: 23.4, PI: 19.5). Bio8 (Mean Temperature of Wettest Quarter) was of minor importance (PC: 4.5, PI: 12.7). The 10^th^ percentile training presence logistic threshold was 0.36 and this value was applied to map and calculate the suitable habitat. The resulting fits were extrapolated to the past climate. According to the global climate model MIROC-ESM, the potentially suitable habitat during the breeding season was found to be largest during the mid Holocene (41 % of the total land area covered in the analysis) and smallest during the Last Glacial Maximum (19 %) (Fig. 4). Large surfaces of suitable habitat were also observed during the last interglacial (37 %) and present (39 %) (Fig. 4). Results obtained with alternative climate models showed similar outputs and are provided in the additional files section (Additional file 7).

## Discussion

We found genetic evidence of historical fluctuations in the effective population size of the turtle dove. As predicted, we inferred a drastic demographic expansion, estimated to have taken place during the early-mid Holocene. Our analyses also supported a relatively recent demographic contraction, which took place between the mid Holocene and the present. The demographic scenario supported by our genetic data is correlated with changes in the availability of suitable habitat for the turtle dove, as inferred by our ecological niche modelling results. We did not, however, detect evidence of population genetic structure associated to the alternative migratory flyways. Finally, we argue that the turtle dove’s fluctuating demography along with its decreasing effective population size make it particularly sensitive to current anthropogenic threats.

### A fluctuating demographic history

The turtle dove is a warm-climate adapted species, with specific breeding habitat requirements that include a mixture of grasslands and woodlands [24]. The climate oscillations that occurred during the Quaternary period strongly affected the distribution and abundance of this kind of environment [47]. Therefore, we expected to find changes in the turtle dove effective population size in response to those shifts.

The last interglacial (~140–120,000 ybp) was a warm period, with climate conditions that were similar to those of the Holocene [48]; therefore, we expected to observe a large *Ne* during that time. Although our niche modelling showed a large surface of suitable turtle dove habitat during this period (Fig. 4), the genetic data did not support our prediction or was not informative about this period in time. We also expected to observe a drastic demographic contraction during the late Pleistocene, when a cooling process started ~75,000 ybp and lasted until the last glacial maximum [49]. This cooling was coupled with shifts in precipitation patterns, creating an east–west gradient [5] and modifying habitat composition [5, 48]. The western Palearctic in general and the northwest Palearctic in particular, became dominated by dry and open-tundra like environments [47], unsuitable for most woodland dependent bird species. In agreement with the described scenario, we found a drastic reduction in the surface of available suitable habitat for the turtle dove (Fig. 4) and a very small estimated *Ne* (Table 1, Fig. 2). It is possible that this period represented a bottleneck for the species. If very few gene trees coalesce prior to the bottleneck, the demographic signal before the last glacial maximum would be erased. Such a bottleneck is a plausible explanation of why our data do not support models that predict a large *Ne* during the last interglacial.

The beginning of the Holocene is characterized by a rise in the average temperatures [50], which generated an increment in the suitable available habitat for most warm-temperature adapted species such as the turtle dove (Fig. 4, [51]). In agreement with this, our Tajima’s *D* and *Fs* analysis suggest a scenario of a recent demographic expansion, which, according to the EBSP analysis started during the early Holocene and reached the highest *Ne* in the present (Fig. 3). Our DIYABC analysis, however, supports a scenario in which the turtle dove went through a strong demographic expansion during the early-mid Holocene and reached its highest *Ne* during the mid Holocene (Fig. 2 and Table 1). This analysis also inferred the occurrence of a recent contraction in the *Ne*, indicating that the current *Ne* for the turtle dove is relatively low and closer to that observed during the late Pleistocene (Table 1). This finding fits our predictions; because it is coherent with the long-term abundance decline reported for the turtle dove [27]. However, our estimate of when this *Ne* contraction started is imprecise and depends on several assumptions, including mutation rates, generation time and the parameters in our model. The observed contraction in the turtle dove effective size could have started at any point between the mid Holocene and the present (Table 1). Therefore, we are unable to assert that it is the strict consequence of modern human activities; moreover it also coincides with a slight reduction in the availability of suitable habitat for the species (projected data, Fig. 4).

Our analyses complement each other, and the observed differences are probably a consequence of the amount of genetic data each method is able to incorporate, its assumptions, and differences in the time scale at which mitochondrial and nuclear markers are able to provide resolution. Nonetheless, all our analyses coincide in showing a history of demographic fluctuations in the turtle dove. Overall, it is likely that this last contraction in the *Ne* is the outcome of the turtle dove’s fluctuating demographic history responding to natural climatic changes, currently enhanced by anthropogenic actions.

### Population genetic structure: different flyways, the same gene pool

We hypothesized that the alternative flyways used by migrating turtle doves between Europe and Africa, would generate genetic differentiation among populations, a phenomenon previously described for other long distance migratory bird species worldwide (e.g. [20, 21, 52]). However, we did not detect any evidence of population genetic structure (Fig. 1b, c and Additional file 4). The turtle doves’ breeding range expands east of Bulgaria, through Russia, reaching China [27]. Therefore we cannot comment on the possibility of genetic structure beyond the three European flyways, that were the focus of our study.

We propose two possible explanations for not finding genetic structure associated to the migratory flyways. 1) The described flyways for the turtle dove are coupled with a weak migratory connectivity (as defined in [53]), meaning that individuals from the same breeding population do not share the same wintering ground and vice versa. Therefore, these flyways would not constitute a barrier to gene flow. 2) Migratory connectivity could be strong, as suggested by the very low probability of recapturing western breeders in the eastern flyway and vice versa [26]. If the latter held true, then flyways could generate genetic differentiation that we might not be able to detect, because divergence is too recent and individuals still share most of their alleles (i.e., high levels of incomplete lineage sorting). Our genetic data will not allow us to distinguish between these alternatives. However, the observation of one individual that was ringed as a western breeder but recaptured on eastern breeding grounds, together with the moderate probability of western and eastern breeders to be recaptured in the central flyway [26] would support the first explanation.

### Implications for conservation

Our results suggest that the turtle dove’s demography make it particularly vulnerable to anthropogenic threats. Firstly, we found that it is undergoing a phase of effective population size contraction (Fig. 2, Table 1). This is of particular interest because the turtle dove has been recently categorized as vulnerable by the IUCN Red List (http://www.iucnredlist.org/), as a consequence of a global abundance decline (30–49 % in 15 years, [27]). This trend has been particularly dramatic in the United Kingdom (97 % decline in 40 years, [54]). The reported decline is attributed to anthropogenic degradation of its breeding [55] and wintering territories [56], also game hunting and poaching [57, 58]. Even though we cannot attribute our finding of a recent *Ne* contraction to modern human actions alone (discussed above), this is still relevant to turtle dove’s conservation. Many bird species that also suffered recent declines in abundance and are categorized as endangered (sensu IUCN Red List) show genomic signatures of long-term demographic declines that predate the appearance of modern anthropogenic threats [59]. We also found signatures of a demographic history of fluctuations for the turtle dove. It has been observed that in demographically stable wild species the ratio of the effective and census population size (*Ne*/*Nc*) is around 0.1 [60], however several factors can produce deviations from this ratio [61]. The estimated *Nc* for the turtle dove in Europe is between 6 and 12 million individuals [27] and our mean *Ne* estimate for the present is ~172,000 individuals (Table 1). Using the latter *Nc* and *Ne* parameters we observe a lower ratio than expected, *Ne*/*Nc* = 0.028–0.014, which is usually the result of drastic demographic fluctuations [62, 63]. Because factors such as sampling bias might affect the estimation of demographic parameters obtained using different analytical methods (e.g. [64]), we suggest to interpret our demographic parameters with caution and instead to focus on the general pattern. In this regard, species that exhibit the described drastic natural fluctuations could be particularly sensitive to anthropogenic threats, especially when they are going through a contraction phase (e.g. North American passenger pigeon, *Ectopistes migratorius,* [65], and Rocky Mountain grasshopper, *Melanoplus spretus*, [66]).

The described demographic scenario (i.e. long-term decline and drastic fluctuations) could increase species’ vulnerability to the impacts of macroecological changes resulting from natural earth cycles, which are currently enhanced by anthropogenic activities [67, 68]. To further illustrate this, a paradigmatic case is the North American passenger pigeon; which went extinct almost a century ago, although it was considered one of the most abundant bird species in the world [69]. A genomic study of this species suggested that its naturally fluctuating demographic history, coupled with a downward trend in its *Ne* that coincided with the appearance of human threats were the main causes of its sudden extinction [65].

## Conclusions

Our finding that the turtle dove shows the genomic signatures of a fluctuating demographic history coupled with a recent effective population size contraction, provides support for its vulnerable conservation status. Finally, because we found no evidence of population genetic structure, we prompt that all turtle dove populations across Europe (the portion of the range that was sampled) are equally relevant for conservation.

## Additional files

Additional file 1: Details of the samples used. A table with additional information on the blood and tissue samples obtained for our work. (DOC 143 kb)
Additional file 2: Further details on materials and methods. Additional information on the laboratory methods related to the RAD-seq procedures and mtDNA sequencing. Also, further details on the demographic history modeling performed with DIYABC, and the niche modeling analysis conducted in MAXENT. (DOC 30 kb)
Additional file 3: Analysis of molecular variance (AMOVAs). Results of the AMOVAs performed, testing for significant genetic differences among the three main flyways (using mitochondrial and nuclear DNA data). (DOC 41 kb)
Additional file 4: fastSTRUCTURE analysis indicating the number of genetic clusters suggested for the turtle dove, *K* = 1. Barplot graphics representing *K* = 1 and 3 are shown. (DOC 393 kb)
Additional file 5: Principal components analysis to test the goodness-of-fit of each evaluated scenario against the simulated data in DIYABC. PCA plots show that Scenario 4 had the best goodness-of-fit among all tested scenarios. (DOC 630 kb)
Additional file 6: Posterior probability of scenario choice in DIYABC. Direct estimation approach that uses 0.1 % of the closest simulations to the observed data. (b) The logistic regression uses 1 % of the closest simulations. (DOC 211 kb)
Additional file 7:
MaxEnt habitat suitability models for European Turtle doves. Niche modelling analysis based on additional climate models: CC (CCSM4), MR (MIROC-ESM) and MI (MPI-ESM-P). Models available on http://www.worldclim.org/. (DOC 729 kb)
