# Supplementary material for: Genomic evidence of demographic fluctuations and lack of genetic structure across flyways in a long distance migrant, the European turtle dove
Source: BMC Evol Biol. 2016 Nov 7;16:237. doi: 10.1186/s12862-016-0817-7 (PMC5100323; doi:10.1186/s12862-016-0817-7)
Supplement: Additional file 1: — Details of the samples used. A table with additional information on the blood and tissue samples obtained for our work. (DOC 143 kb) [file 12862_2016_817_MOESM1_ESM.doc]

Additional file 1. Details of the samples used for this study.

| Sample ID | Locality | Country | Ring ID | Lat. | Long. | Sample type | Sample timing | Year |
| --- | --- | --- | --- | --- | --- | --- | --- | --- |
| UK_003 | Essex | United Kingdom | EG59012 | 51.76 | 0.47 | Blood-EtOH | Breeding | 2011 |
| UK_005 | Essex | United Kingdom | EG59014 | 51.76 | 0.47 | Blood-EtOH | Breeding | 2011 |
| UK_008 | Cambridgeshire | United Kingdom | EG59024 | 52.2 | 0.12 | Blood-EtOH | Breeding | 2011 |
| UK_009 | Essex | United Kingdom | EG59028 | 51.76 | 0.47 | Blood-EtOH | Breeding | 2012 |
| UK_010 | Essex | United Kingdom | EG59029 | 51.76 | 0.47 | Blood-EtOH | Breeding | 2012 |
| UK_011 | Essex | United Kingdom | EG59030 | 51.76 | 0.47 | Blood-EtOH | Breeding | 2012 |
| UK_012 | Essex | United Kingdom | EG59031 | 51.76 | 0.47 | Blood-EtOH | Breeding | 2012 |
| UK_013 | Essex | United Kingdom | EG59033 | 51.76 | 0.47 | Blood-EtOH | Breeding | 2012 |
| UK_014 | Norfolk | United Kingdom | EG59037 | 52.56 | 0.34 | Blood-EtOH | Breeding | 2012 |
| UK_015 | Essex | United Kingdom | EG59039 | 51.76 | 0.47 | Blood-EtOH | Breeding | 2012 |
| UK_016 | Essex | United Kingdom | EG59042 | 51.76 | 0.47 | Blood-EtOH | Breeding | 2012 |
| UK_017 | Essex | United Kingdom | EG82557 | 51.76 | 0.47 | Blood-EtOH | Breeding | 2012 |
| UK_018 | Essex | United Kingdom | EG82556 | 51.76 | 0.47 | Blood-EtOH | Breeding | 2012 |
| UK_019 | Essex | United Kingdom | EG59050 | 51.76 | 0.47 | Blood-EtOH | Breeding | 2012 |
| UK_021 | Essex | United Kingdom | EG82563 | 51.76 | 0.47 | Blood-EtOH | Breeding | 2012 |
| UK_022 | Essex | United Kingdom | EG59102 | 51.76 | 0.47 | Blood-EtOH | Breeding | 2013 |
| UK_023 | Essex | United Kingdom | EG59105 | 51.76 | 0.47 | Blood-EtOH | Breeding | 2013 |
| UK_025 | Essex | United Kingdom | EG59111 | 51.76 | 0.47 | Blood-EtOH | Breeding | 2013 |
| FR_074 | Pitou-Charente | France | GY103807 | 46.13 | -0.41 | Blood-FTA | Breeding | 2013 |
| FR_075 | Pitou-Charente | France | GY79894 | 46.13 | -0.41 | Blood-FTA | Breeding | 2013 |
| FR_076 | Pitou-Charente | France | GY120580 | 46.13 | -0.41 | Blood-FTA | Breeding | 2013 |
| FR_077 | Pitou-Charente | France | GY69351 | 46.13 | -0.41 | Blood-FTA | Breeding | 2013 |
| FR_078 | Pitou-Charente | France | GY69576 | 46.13 | -0.41 | Blood-FTA | Breeding | 2013 |
| FR_080 | Pitou-Charente | France | GY103809 | 46.13 | -0.41 | Blood-FTA | Breeding | 2013 |
| FR_081 | Pitou-Charente | France | GY103810 | 46.13 | -0.41 | Blood-FTA | Breeding | 2013 |
| FR_294 | Auvergne | France | GY119763 | 45.07 | 3.34 | Blood-FTA | Breeding | 2014 |
| FR_295 | Auvergne | France | GY119764 | 45.07 | 3.34 | Blood-FTA | Breeding | 2014 |
| FR_296 | Auvergne | France | GY119765 | 45.07 | 3.34 | Blood-FTA | Breeding | 2014 |
| FR_297 | Auvergne | France | GY119766 | 45.07 | 3.34 | Blood-FTA | Breeding | 2014 |
| FR_301 | Marne | France | GY119652 | 49.07 | 4.13 | Blood-FTA | Breeding | 2014 |
| FR_302 | Marne | France | GY119653 | 49.07 | 4.13 | Blood-FTA | Breeding | 2014 |
| FR_303 | Marne | France | GY119685 | 49.07 | 4.13 | Blood-FTA | Breeding | 2014 |
| FR_304 | Marne | France | GY119660 | 49.07 | 4.13 | Blood-FTA | Breeding | 2014 |
| FR_305 | Marne | France | GY119664 | 49.07 | 4.13 | Blood-FTA | Breeding | 2014 |
| FR_306 | Marne | France | GY119667 | 49.07 | 4.13 | Blood-FTA | Breeding | 2014 |
| FR_307 | Marne | France | GY119668 | 49.07 | 4.13 | Blood-FTA | Breeding | 2014 |
| FR_308 | Marne | France | GY119670 | 49.07 | 4.13 | Blood-FTA | Breeding | 2014 |
| SP_212 | Monfrague | Spain | - | 39.81 | -5.93 | Tissue | Breeding | 2013 |
| SP_213 | Monfrague | Spain | - | 39.81 | -5.93 | Tissue | Breeding | 2013 |
| SP_214 | Monfrague | Spain | - | 39.81 | -5.93 | Tissue | Breeding | 2013 |
| SP_236 | Monfrague | Spain | - | 39.81 | -5.93 | Tissue | Breeding | 2013 |
| SP_240 | Monfrague | Spain | - | 39.81 | -5.93 | Tissue | Breeding | 2013 |
| SP_245 | Monfrague | Spain | - | 39.81 | -5.93 | Tissue | Breeding | 2013 |
| SP_246 | Monfrague | Spain | - | 39.81 | -5.93 | Tissue | Breeding | 2013 |
| SP_247 | Monfrague | Spain | - | 39.81 | -5.93 | Tissue | Breeding | 2013 |
| SP_249 | Monfrague | Spain | - | 39.81 | -5.93 | Tissue | Breeding | 2013 |
| SP_250 | Monfrague | Spain | - | 39.81 | -5.93 | Tissue | Breeding | 2013 |
| IT_167 | Isla Ventotene | Italy | H196179 | 40.79 | 13.43 | Blood-FTA | Migrating | 2014 |
| IT_168 | Isla Ventotene | Italy | H196180 | 40.79 | 13.43 | Blood-FTA | Migrating | 2014 |
| IT_169 | Isla Ventotene | Italy | H196181 | 40.79 | 13.43 | Blood-FTA | Migrating | 2014 |
| IT_170 | Isla Ventotene | Italy | H196182 | 40.79 | 13.43 | Blood-FTA | Migrating | 2014 |
| IT_171 | Isla Ventotene | Italy | H196183 | 40.79 | 13.43 | Blood-FTA | Migrating | 2014 |
| IT_172 | Isla Ventotene | Italy | H196184 | 40.79 | 13.43 | Blood-FTA | Migrating | 2014 |
| IT_173 | Isla Ventotene | Italy | H196185 | 40.79 | 13.43 | Blood-FTA | Migrating | 2014 |
| IT_174 | Isla Ventotene | Italy | H196186 | 40.79 | 13.43 | Blood-FTA | Migrating | 2014 |
| IT_175 | Isla Ventotene | Italy | H196187 | 40.79 | 13.43 | Blood-FTA | Migrating | 2014 |
| IT_176 | Isla Ventotene | Italy | H196191 | 40.79 | 13.43 | Blood-FTA | Migrating | 2014 |
| IT_177 | Isla Ventotene | Italy | H196189 | 40.79 | 13.43 | Blood-FTA | Migrating | 2014 |
| IT_178 | Isla Ventotene | Italy | H196188 | 40.79 | 13.43 | Blood-FTA | Migrating | 2014 |
| IT_180 | Isla Ventotene | Italy | H196194 | 40.79 | 13.43 | Blood-FTA | Migrating | 2014 |
| IT_181 | Isla Ventotene | Italy | H196195 | 40.79 | 13.43 | Blood-FTA | Migrating | 2014 |
| IT_182 | Isla Ventotene | Italy | H196202 | 40.79 | 13.43 | Blood-FTA | Migrating | 2014 |
| MA_253 | Comino | Malta | - | 36 | 14.33 | Tissue | Migrating | 2013 |
| MA_255 | Comino | Malta | - | 36 | 14.33 | Tissue | Migrating | 2013 |
| MA_312 | Comino | Malta | - | 36 | 14.33 | Tissue | Migrating | 2014 |
| MA_313 | Comino | Malta | - | 36 | 14.33 | Tissue | Migrating | 2014 |
| MA_315 | Comino | Malta | - | 36 | 14.33 | Tissue | Migrating | 2014 |
| MA_316 | Comino | Malta | DD1191 | 36 | 14.33 | Blood-FTA | Migrating | 2014 |
| MA_317 | Comino | Malta | DD1192 | 36 | 14.33 | Blood-FTA | Migrating | 2014 |
| MA_318 | Comino | Malta | DD1193 | 36 | 14.33 | Blood-FTA | Migrating | 2014 |
| MA_319 | Comino | Malta | EE02043 | 36 | 14.33 | Blood-FTA | Migrating | 2014 |
| MA_320 | Comino | Malta | EE02044 | 36 | 14.33 | Blood-FTA | Migrating | 2014 |
| MA_321 | Comino | Malta | EE02045 | 36 | 14.33 | Blood-FTA | Migrating | 2014 |
| GE_311 | Hesse | Germany | - | 50.55 | 8.69 | Blood-FTA | Breeding | 2014 |
| BU_052 | Dobrich | Bulgaria | - | 43.44 | 28.33 | Tissue | Breeding | 2014 |
| BU_053 | Dobrich | Bulgaria | - | 43.44 | 28.33 | Tissue | Breeding | 2014 |
| BU_054 | Dobrich | Bulgaria | - | 43.44 | 28.33 | Tissue | Breeding | 2014 |
| BU_055 | Dobrich | Bulgaria | - | 43.44 | 28.33 | Tissue | Breeding | 2014 |
| BU_057 | Dobrich | Bulgaria | - | 43.44 | 28.33 | Tissue | Breeding | 2014 |
| BU_056 | Dobrich | Bulgaria | - | 43.44 | 28.33 | Tissue | Breeding | 2014 |
| BU_058 | Dobrich | Bulgaria | - | 43.44 | 28.33 | Tissue | Breeding | 2014 |
| BU_059 | Dobrich | Bulgaria | - | 43.44 | 28.33 | Tissue | Breeding | 2014 |
| BU_060 | Dobrich | Bulgaria | - | 43.44 | 28.33 | Tissue | Breeding | 2014 |
| BU_061 | Dobrich | Bulgaria | - | 43.44 | 28.33 | Blood-FTA | Breeding | 2012 |
| BU_062 | Dobrich | Bulgaria | - | 43.44 | 28.33 | Blood-FTA | Breeding | 2012 |
| BU_063 | Dobrich | Bulgaria | - | 43.44 | 28.33 | Blood-FTA | Breeding | 2012 |
| BU_066 | Dobrich | Bulgaria | - | 43.44 | 28.33 | Blood-FTA | Breeding | 2012 |
| BU_067 | Dobrich | Bulgaria | - | 43.44 | 28.33 | Blood-FTA | Breeding | 2012 |
| GR_117 | Evros | Greece | - | 41.19 | 26.24 | Tissue | Breeding/Mig. | 2013 |
| GR_118 | Evros | Greece | - | 41.19 | 26.24 | Tissue | Breeding/Mig. | 2013 |
| GR_119 | Evros | Greece | - | 41.19 | 26.24 | Tissue | Breeding/Mig. | 2013 |
| GR_122 | Evros | Greece | - | 41.19 | 26.24 | Tissue | Breeding/Mig. | 2013 |
| GR_124 | Evros | Greece | - | 41.19 | 26.24 | Tissue | Breeding/Mig. | 2013 |
| GR_126 | Evros | Greece | - | 41.19 | 26.24 | Tissue | Breeding/Mig. | 2013 |
| GR_133 | Evros | Greece | - | 41.19 | 26.24 | Tissue | Breeding/Mig. | 2013 |
| GR_136 | Evros | Greece | - | 41.19 | 26.24 | Tissue | Breeding/Mig. | 2013 |
| GR_137 | Evros | Greece | - | 41.19 | 26.24 | Tissue | Breeding/Mig. | 2013 |
| GR_141 | Evros | Greece | - | 41.19 | 26.24 | Tissue | Breeding/Mig. | 2013 |
| GR_143 | Evros | Greece | - | 41.19 | 26.24 | Tissue | Breeding/Mig. | 2013 |
| GR_147 | Evros | Greece | - | 41.19 | 26.24 | Tissue | Breeding/Mig. | 2013 |
| GR_148 | Evros | Greece | - | 41.19 | 26.24 | Tissue | Breeding/Mig. | 2013 |
| GR_149 | Evros | Greece | - | 41.19 | 26.24 | Tissue | Breeding/Mig. | 2013 |
| GR_150 | Evros | Greece | - | 41.19 | 26.24 | Tissue | Breeding/Mig. | 2013 |
