# Supplementary material for: Genomic evidence of demographic fluctuations and lack of genetic structure across flyways in a long distance migrant, the European turtle dove
Source: BMC Evol Biol. 2016 Nov 7;16:237. doi: 10.1186/s12862-016-0817-7 (PMC5100323; doi:10.1186/s12862-016-0817-7)
Supplement: Additional file 2: — Further details on materials and methods. Additional information on the laboratory methods related to the RAD-seq procedures and mtDNA sequencing. Also, further details on the demographic history modeling performed with DIYABC, and the niche modeling analysis conducted in MAXENT. (DOC 30 kb) [file 12862_2016_817_MOESM2_ESM.doc]

Additional file 2. Further details on materials and methods section.

*Laboratory methods:*

*- ddRAD-seq*: A total of ~500 ng of DNA per sample were digested using two restriction enzymes: SbfI High Fidelity and MspI (New England Biolabs, MA). Digested products were ligated to 5’ P1 and 3’ P2 adapters, the former contained unique 5-7 bp barcodes for multiplexing. We pooled samples with unique P1 barcodes into six different indexing groups and size selected fragments between 400-700 bp using Blue Pippin (Sage Science, MA). For each indexing group we incorporated Illumina TruSeq adapters by performing a low number of PCR cycles. The six indexing groups were combined in equimolar proportions into two final libraries, each sequenced on an Illumina HiSeq 2500 lane at the Cornell University Institute for Biotechnology, obtaining single-end 151 bp sequences.

*- mtDNA:* PCRs were conducted in a reaction volume of 20 μl containing 100 ng of DNA template, 10 mM of each primer, 10 mM dNTPs (Roth, Karlsruhe), 2 mM MgCl, 5 U *Thermus aquaticus* polymerase (BioLabs TaqDNA polymerase) and 1x PCR buffer. Thermocycling included an initial denaturation at 94°C for 2 min, 35 cycles of denaturation at 94°C for 30 s, annealing at 58°C for 1 min and extension at 72°C for 1 min, followed by a final extension step of 5 min at 72°C. Products were purified of excess primers and dNTPs using exonuclease-shrimp alkaline phosphatase (Fermentas Life Sciences; following the manufacturer’s specifications). PCR products were then sequenced in both directions using Big Dye chemistry (Applied Biosystems) and run on an ABI 3130xl genetic analyzer (Applied Biosystems). Resulting sequences were assembled and aligned in CLC Main Workbench 6.9.2.

*Demographic history and niche modelling:*

*- Details on the five evaluated demographic scenarios in DIYABC:* Scenario #1 resembles the demographic scenario inferred by the EBSP analysis, with a small effective size during the late Pleistocene (LP) and a demographic expansion, which started at the beginning of the Holocene (H), 14,000 ybp. Scenario #2 builds on scenario 1 by including a demographic expansion that occurred during the last interglacial (LIG), with an *Ne* equal to the one reached during Holocene, as consequence of similar amounts of available suitable habitat during both periods (figure 4). Scenario #3 builds on #2 by proposing a demographic reduction that occurred somewhere between the present and the beginning of the Holocene. This contraction in the *Ne* is based on the drastic demographic decline reported for the species. Scenario #4 considers a small *Ne* during the mid-late Pleistocene, a demographic expansion that occurred at the beginning of the Holocene and a posterior contraction. Finally, scenario #5 assumes a constant effective population size through time.

*- Niche modelling*: The realized niche of the present distribution was modelled and extrapolated using the Bioclim data set and MaxEnt 3.3.3k (maximum entropy; [1]), a program for modelling ecological niches from presence-only species records. Presence data were obtained from GBIF (Global Biodiversity Information Facility, <http://www.gbif.org/>), as GBIF Occurrence Download: (24th September 2015), DOI: <http://doi.org/10.15468/dl.gqbvoj>), using the search criteria: *Taxon: Streptopelia turtur (Linnaeus, 1758)*, *Georeferenced: true*. This produced a database of 56,602 occurrence records, which originated from 233 datasets. Data from countries outside the breeding area or records collected before 1950 were removed. Along the western and central flyway, some countries were over-represented (UK, Netherlands, Belgium, Germany, Austria). Therefore, we calculated the expected number of observations relative to the mean population size according to Tucker et al. (1994), based on the number of observations for France (n=3,630). The calculated number of observations was then selected randomly from the data of the countries, providing a more homogenous data set of 17,424 occurrence records. From the Bioclim database of downscaled climate data from simulations with Global Climate Models (GCMs), we downloaded (<http://worldclim.org/download>) the following sets of climate data at a spatial resolution of 2.5 arcmin (about 4 km at the equator) in GeoTIFF format: present data, data for the Mid-Holocene (MI, 6000 years ago) and the Last Glacial Maximum (LGM, 22,000 years ago), as well as the data for the last interglacial (LIG; ~120,000 - 140,000 years BP) at 30 arc-seconds resolution (ca. 900m at the equator). Three sets of MI and LGM data were downloaded based on three Global Climate Models (GCMs): CCSM4, MIROC-ESM and MPI-ESM-P. Three models based on these different GCMs were run for the simulation of past habitat suitability as recommended [2], but as these produced similar results, only MIROC-ESM results are shown in the main text (figure 4), with the other models provided as additional files (Additional file 7). The GeoTIFF files were opened in ArcGIS 10.2. (ESRI, Redlands, CA, USA) and converted to ASCII format. Using DIVA-GIS 7.4.0.1 (available from http://www.diva-gis.org), we extracted (‘extract values by points’) the 19 Bioclim variables and calculated a correlation matrix. This matrix was used to select redundant variables, which were not included in the MaxEnt analysis. The four remaining variables were: Bio1 (Annual mean temperature), Bio8 (Mean Temperature of Wettest Quarter), Bio12 (Annual Precipitation) and Bio18 (Precipitation of Warmest Quarter). The grids of these variables were trimmed to an area of latitudes 30°N to 60°N and longitudes 30°W to 30°E. Although the breeding area extends further east, Eastern Europe and Russia are poorly represented in the GBIF database. By trimming at 30°E we limited the presence of these underrepresented areas in the model and thus reduced the bias.

The MaxEnt program was run with trimmed datasets (ASCII files) of the four non-redundant variables and the following settings: logistic output format, resulting in values between 0 and 1 for each grid cell, where higher values indicate more similar climatic conditions; remove duplicates; and 50 replicate runs of random (bootstrap) sub-samples with 30% random test percentage. The results were summarized as the average of the 50 models, and the area under the receiver operating characteristic curve (AUC) was used for model evaluation. MaxEnt ASCII outputs were imported into DIVA-GIS, and values below the 10th percentile training presence logistic threshold were removed. The projected suitable habitat was then imported into ArcGIS. To estimate variable response, we used the jack-knife approach of MaxEnt.

**Supplementary references**

1- Phillips SJ, Anderson RP, Schapire RE (2006). Maximum entropy modeling of species geographic distributions. *Ecol. Model.* **190**, 231-259.

2- Varela S, Lima-Ribeiro MS, Terribile LC (2015). A short guide to the climatic variables of the last glacial maximum for biogeographers. *PLoS ONE* **10,** e0129037
