# Supplementary material for: Genomic evidence of demographic fluctuations and lack of genetic structure across flyways in a long distance migrant, the European turtle dove
Source: BMC Evol Biol. 2016 Nov 7;16:237. doi: 10.1186/s12862-016-0817-7 (PMC5100323; doi:10.1186/s12862-016-0817-7)
Supplement: Additional file 3: — Analysis of molecular variance (AMOVAs). Results of the AMOVAs performed, testing for significant genetic differences among the three main flyways (using mitochondrial and nuclear DNA data). (DOC 41 kb) [file 12862_2016_817_MOESM3_ESM.doc]

Additional file 3. Analyses of the molecular variance. We tested for significant genetic differences among the three main flyways. Based on (a) mtDNA and (b) nuDNA data.

**(a)**

| Source of Var. | d.f. | Sum of squares | Variance components | % of variation |
| --- | --- | --- | --- | --- |
| Among groups | 2 | 240.245 | -10.26 Va | -406.76 |
| Among pop. within groups | 3 | 6.426 | 6.15 Vb | 244.02 |
| Within pop. | 34 | 225.454 | 6.63 Vc | 262.74 |
| Total | 39 | 472.125 | 2.52 |  |

| Fsc = 0.48 (p=0.34) |
| --- |
| Fst = -1.62 (p=0.74) |
| Fct = -4.06 (p=0.80) |

**(b)**

| Source of Var. | d.f. | Sum of squares | Variance components | % of variation |
| --- | --- | --- | --- | --- |
| Among groups | 2 | 201.4 | -0.41 Va | -0.14 |
| Among pop. within groups | 5 | 541.46 | 0.31 Vb | 0.32 |
| Within pop. | 200 | 20144.8 | 100.7 Vc | 99.8 |
| Total | 207 | 20887.7 | 100.9 |  |

| Fsc = 0.003 (p=0.17) |
| --- |
| Fst = 0.001 (p=0.38) |
| Fct = -0.001 (p=0.89) |
