# Supplementary material for: Genomic evidence of demographic fluctuations and lack of genetic structure across flyways in a long distance migrant, the European turtle dove
Source: BMC Evol Biol. 2016 Nov 7;16:237. doi: 10.1186/s12862-016-0817-7 (PMC5100323; doi:10.1186/s12862-016-0817-7)
Supplement: Additional file 4: — fastSTRUCTURE analysis indicating the number of genetic clusters suggested for the turtle dove, K = 1. Barplot graphics representing K = 1 and 3 are shown. (DOC 393 kb) [file 12862_2016_817_MOESM4_ESM.doc]

Additional file 4. fastSTRUCTURE analysis suggested *K* = 1, also barplot for *K* = 3 is shown. Vertical bars show individuals *Q* values (i.e., probability of belonging to a genetic cluster). Individuals appear grouped according to their sampling origin.
