# Supplementary material for: Genomic evidence of demographic fluctuations and lack of genetic structure across flyways in a long distance migrant, the European turtle dove
Source: BMC Evol Biol. 2016 Nov 7;16:237. doi: 10.1186/s12862-016-0817-7 (PMC5100323; doi:10.1186/s12862-016-0817-7)

Additional file 5. Principal component analysis to test the goodness-of-fit of each evaluated scenario against the simulated data in DIYABC. Scenario 4 had the best goodness-of-fit.


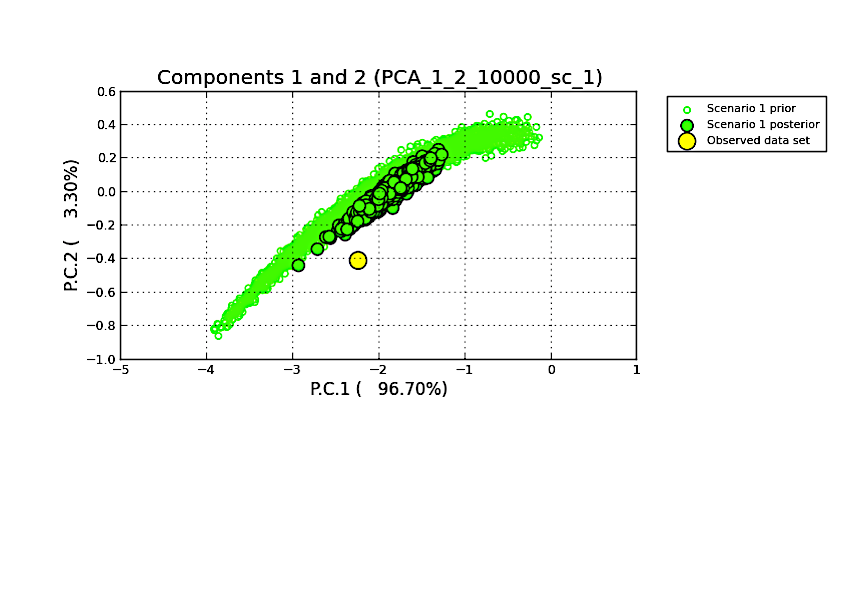


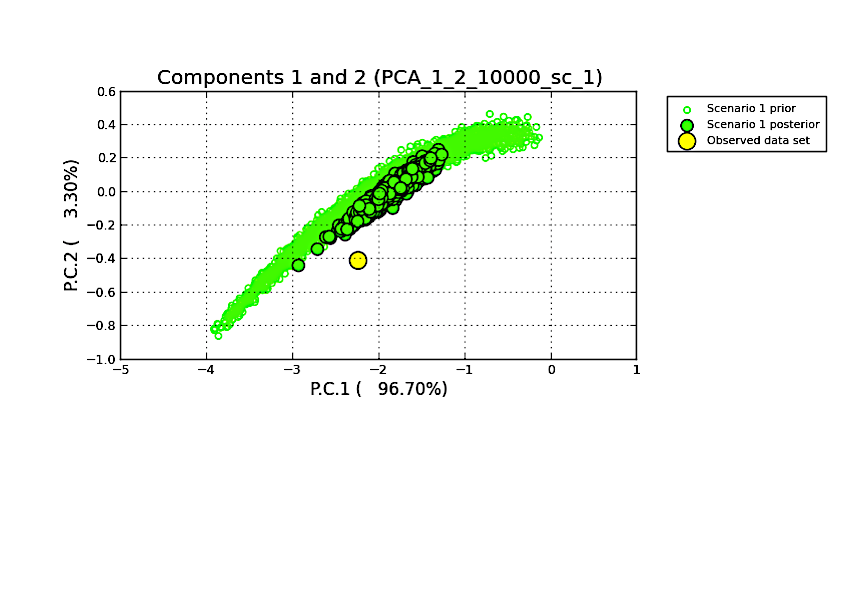


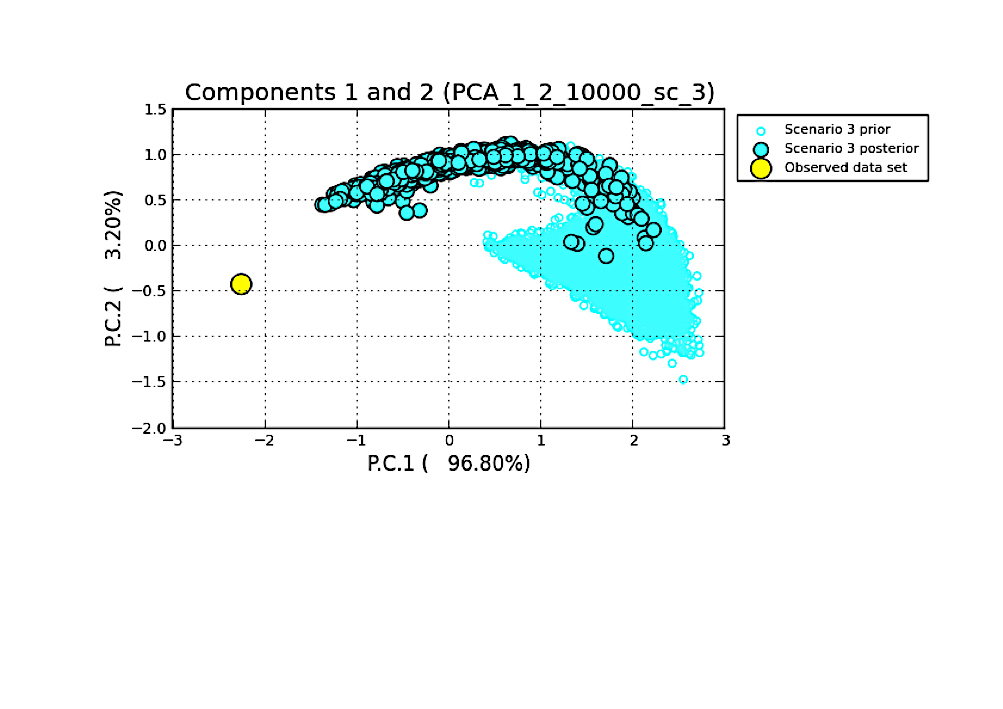

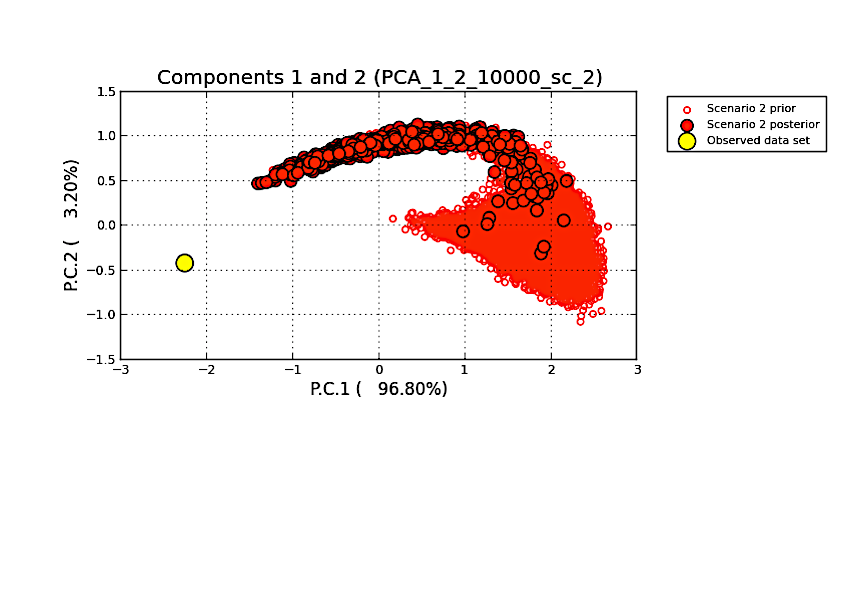

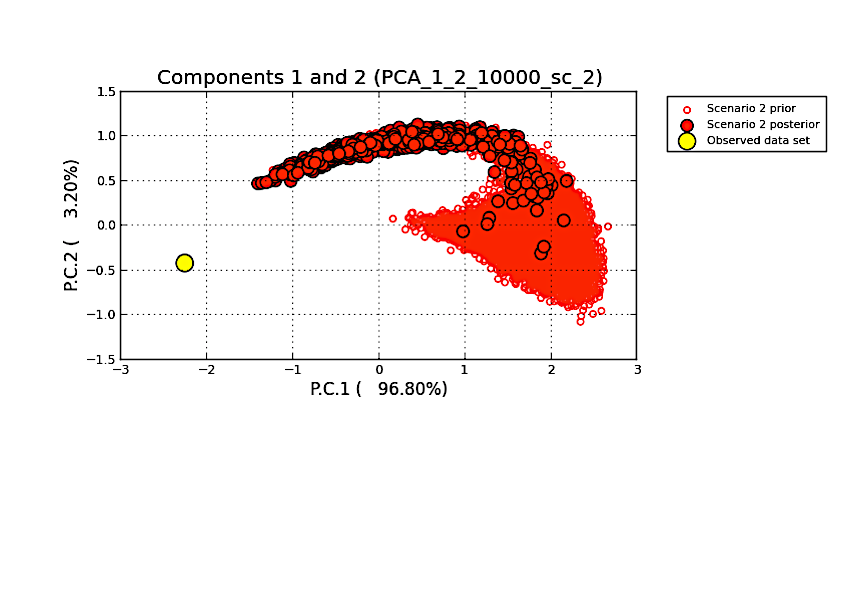

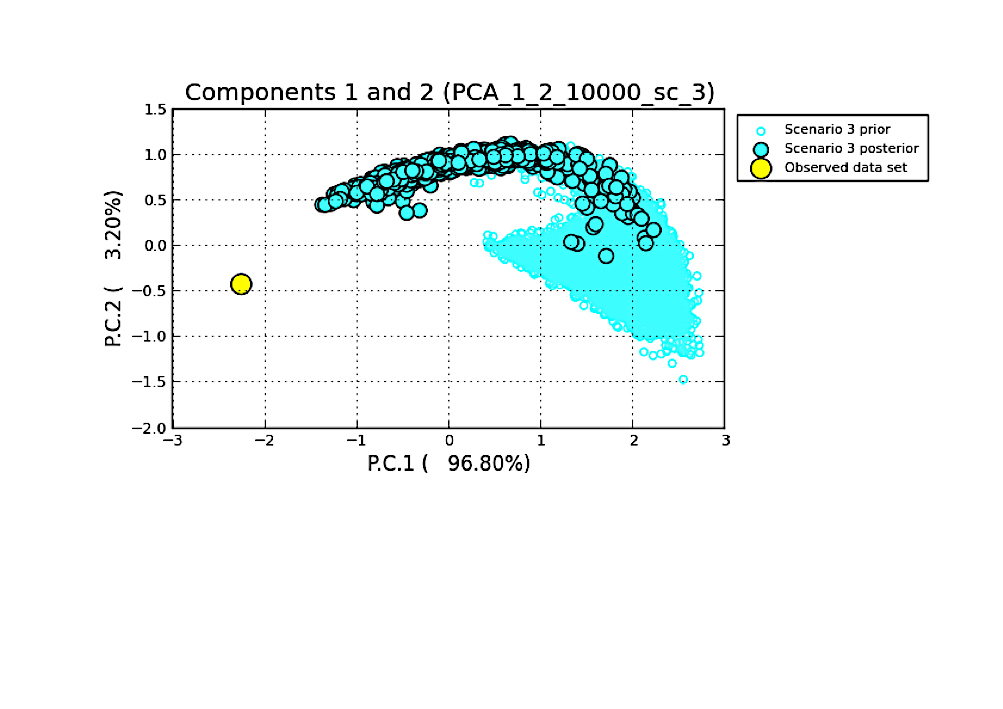


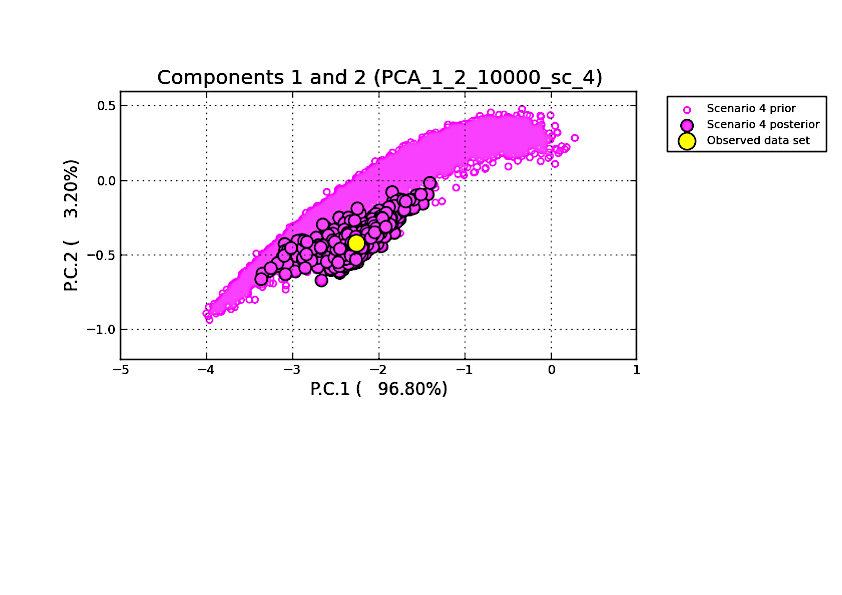

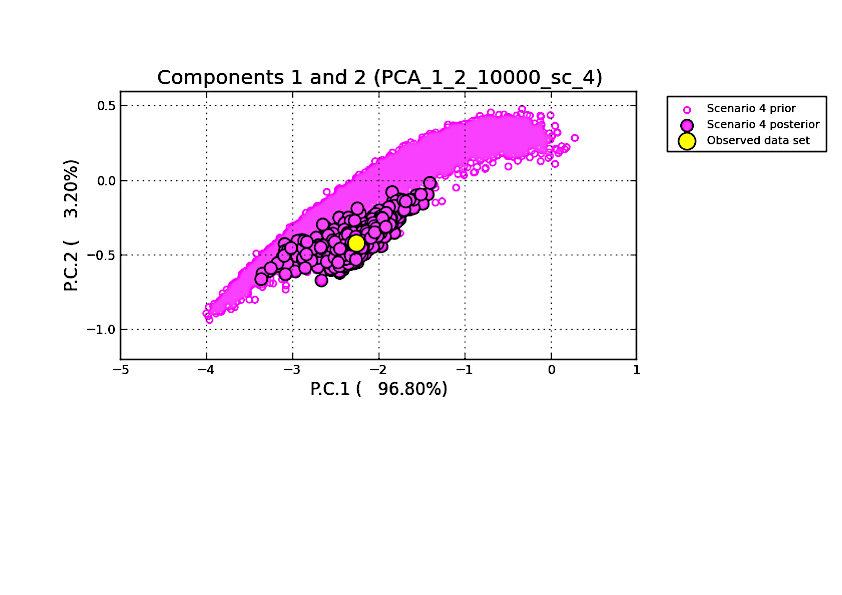

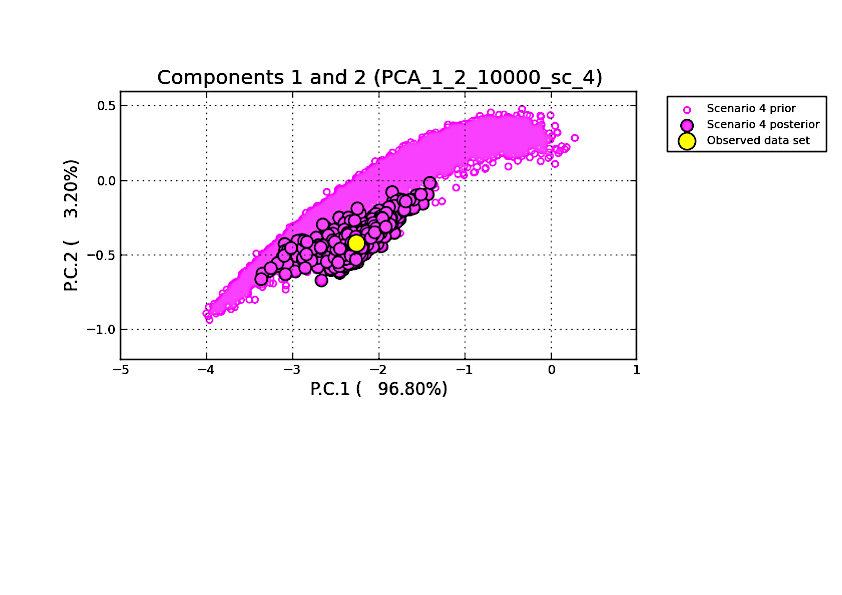


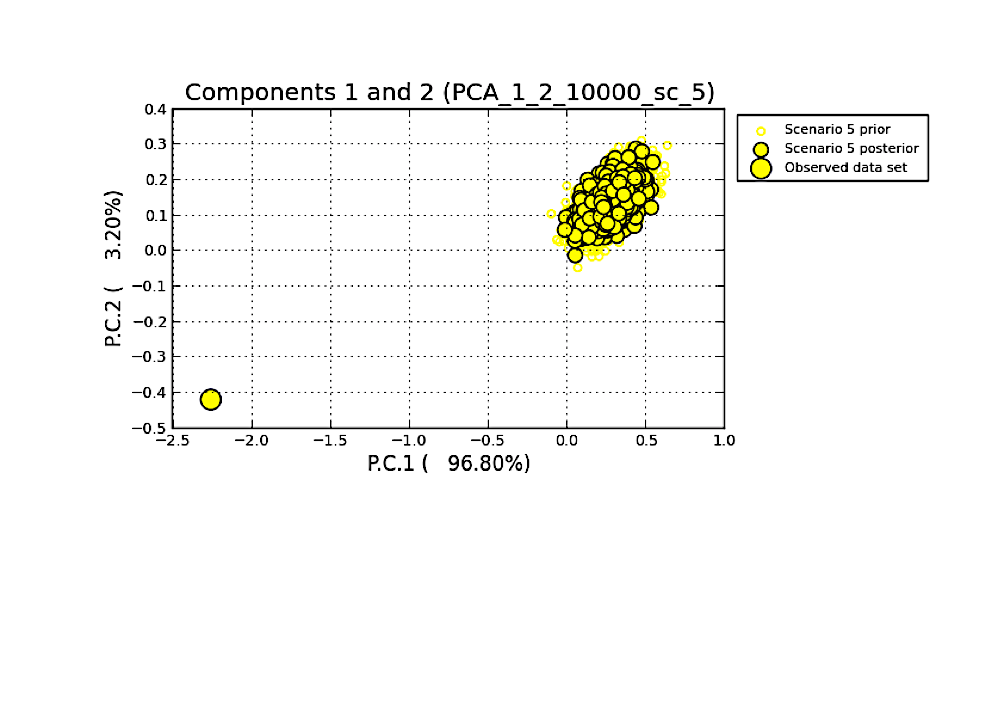

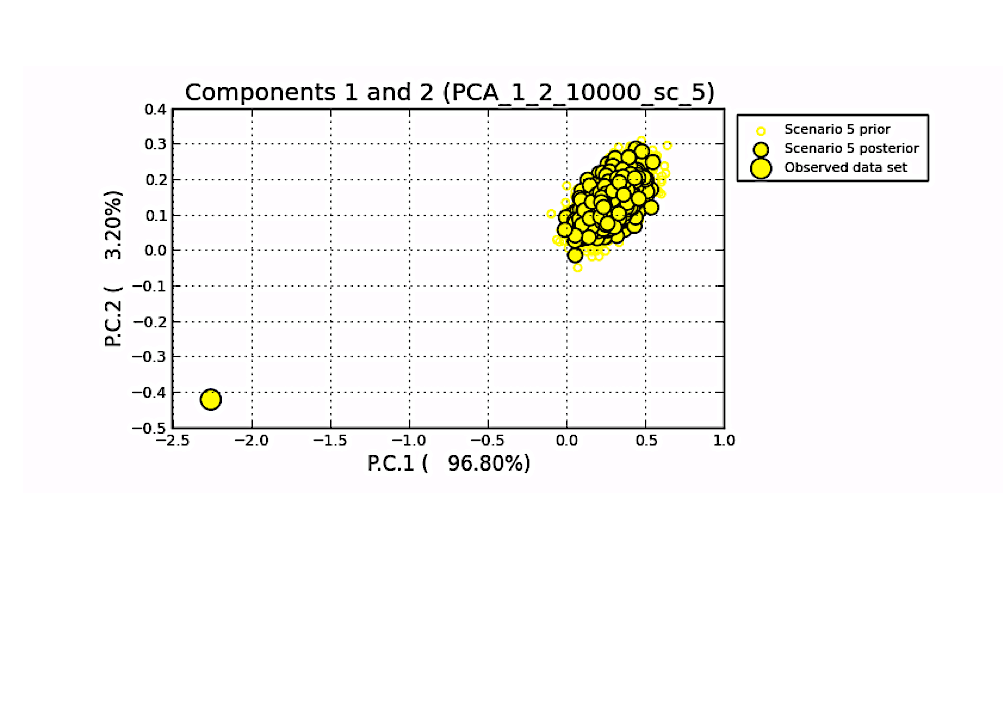

Supplement: Additional file 5: — Principal components analysis to test the goodness-of-fit of each evaluated scenario against the simulated data in DIYABC. PCA plots show that Scenario 4 had the best goodness-of-fit among all tested scenarios. (DOC 630 kb) [file 12862_2016_817_MOESM5_ESM.doc]
