# Supplementary material for: Genomic evidence of demographic fluctuations and lack of genetic structure across flyways in a long distance migrant, the European turtle dove
Source: BMC Evol Biol. 2016 Nov 7;16:237. doi: 10.1186/s12862-016-0817-7 (PMC5100323; doi:10.1186/s12862-016-0817-7)
Supplement: Additional file 6: — Posterior probability of scenario choice in DIYABC. Direct estimation approach that uses 0.1 % of the closest simulations to the observed data. (b) The logistic regression uses 1 % of the closest simulations. (DOC 211 kb) [file 12862_2016_817_MOESM6_ESM.doc]

Additional file 6. Posterior probability of scenario choice. The red line represent scenario #4 and the black lines represent the remaining scenarios. (a) The direct estimation approach uses 0.1% of the closest simulations to the observed data. (b) The logistic regression uses 1% of the closest simulations.
