# Supplementary material for: Genomic evidence of demographic fluctuations and lack of genetic structure across flyways in a long distance migrant, the European turtle dove
Source: BMC Evol Biol. 2016 Nov 7;16:237. doi: 10.1186/s12862-016-0817-7 (PMC5100323; doi:10.1186/s12862-016-0817-7)

Additional file 7. MaxEnt habitat suitability models for European Turtle Doves. The Models for the Mid-Holocene (MI, 6000 years ago) and the Last Glacial Maximum (LGM, 22,000 years ago) were based on three Global Climate Models available on http://www.worldclim.org/paleo-climate: CC (CCSM4), ME (MPI-ESM-P) and MR (MIROC-ESM),


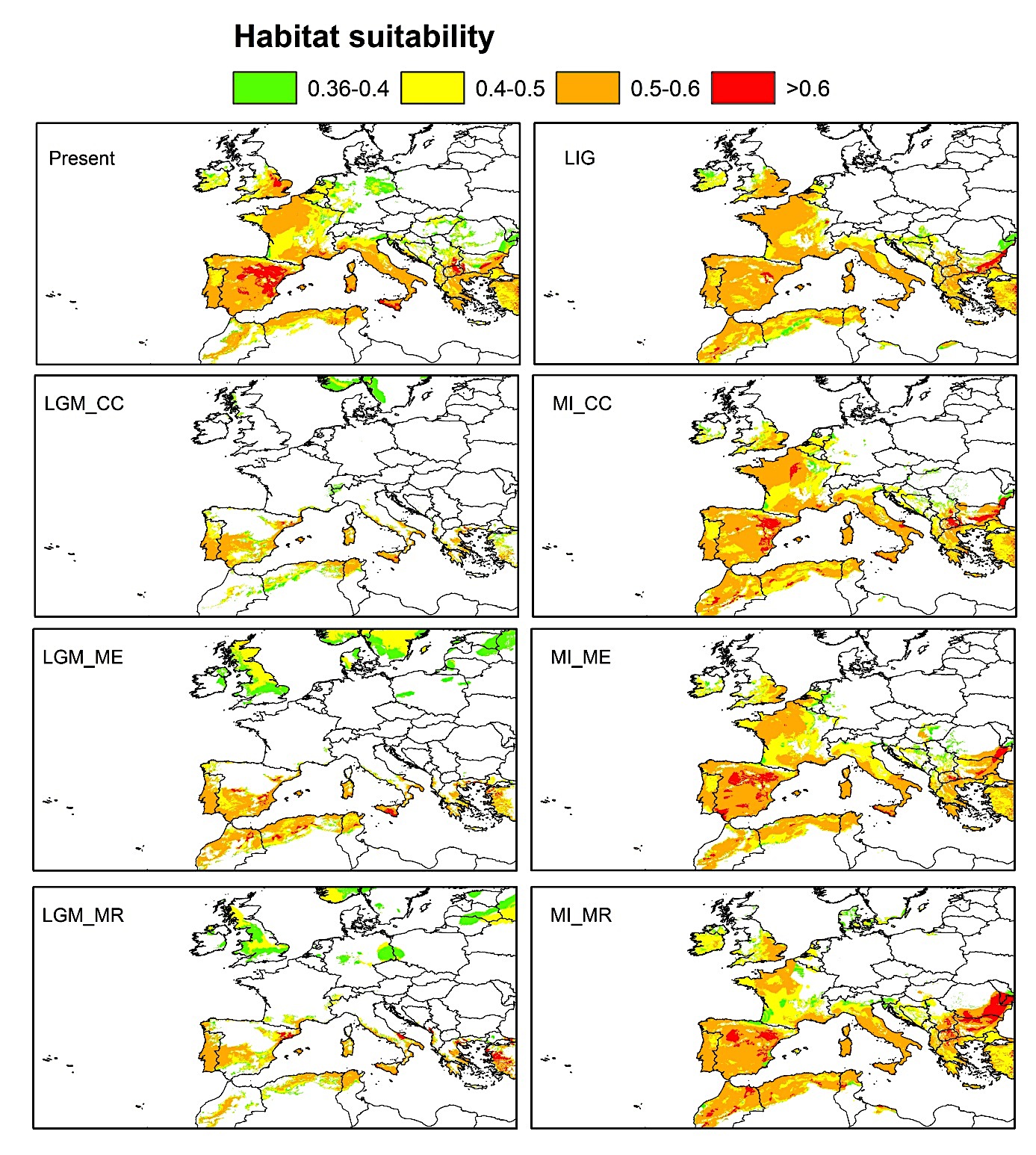

Supplement: Additional file 7: — MaxEnt habitat suitability models for European Turtle doves. Niche modelling analysis based on additional climate models: CC (CCSM4), MR (MIROC-ESM) and MI (MPI-ESM-P). Models available on http://www.worldclim.org/. (DOC 729 kb) [file 12862_2016_817_MOESM7_ESM.doc]
